# 5‐HT_2C_ R antagonist/5‐HT_2C_ R inverse agonist recovered the increased isolation‐induced aggressive behavior of BALB/c mice mediated by ADAR1 (p110) expression and Htr2c RNA editing

**DOI:** 10.1002/brb3.929

**Published:** 2018-02-07

**Authors:** Weizhi Yu, Hong Xu, Ying Xue, Dong An, Huairui Li, Wei Chen, Deqin Yu, Yiping Sun, Jianmei Ma, Yiyuan Tang, Zhaoyang Xiao, Shengming Yin

**Affiliations:** ^1^ College of Basic Medical Sciences Dalian Medical University Dalian China; ^2^ The 2nd Affiliated Hospital Dalian Medical University Dalian China; ^3^ Texas Tech Neuroimaging Institute Texas Tech University Lubbock TX USA

**Keywords:** 5‐HT_2__C_R, ADAR1, aggressive behavior

## Abstract

**Introduction:**

Social isolation enhances the aggressive behavior of animals, but the detailed mechanism remains unclear. Epigenetic studies have suggested that Htr2c RNA editing is closely related to aggressive behavior. This study aims to obtain a fundamental understanding of how social isolation impacts adenosine deaminase acting on RNA 1 (ADAR1, RNA editing enzyme) and Htr2c RNA editing, leading to aggressive behavior, and explore the effective solutions for the recovery of this behavior.

**Methods:**

We evaluated 21‐day‐old BALB/c mice with and without isolation for aggressive behavior using a resident‐intruder test. Immune‐reactivity and protein expression of ADAR1 (p110) were measured using immunohistochemistry and Western blotting. Htr2c RNA editing was evaluated using pyrosequencing. In addition, the 5‐HT_2C_ R antagonist SB243213/5‐HT_2C_ R inverse agonist SB206553 was used to treat the isolated mice, and the performance of both treatments on the behavior, ADAR1 (p110) expression, and Htr2c RNA editing in isolated mice was examined.

**Results:**

Both the protein expression and immune‐reactivity of ADAR1 (p110) in the amygdala decreased, but the percentage of Htr2c RNA editing at A and B sites of amygdala only showed a moderate increase in isolated BALB/c mice with enhanced aggressive behavior compared to the age‐matched group‐housed BALB/c mice. Additionally, treatment with the 5‐HT_2C_ R antagonist SB243213/5‐HT_2C_ R inverse agonist SB206553 recovered the enhanced aggressive behavior of isolated mice and returned the protein expression and immune‐reactivity of ADAR1 (p110) back to the normal level. Moreover, compared to the age‐matched isolated mice treated with physiological saline, isolated mice treated with 5‐HT_2C_ R inverse agonist SB206553 showed a lower percentage of Htr2c RNA editing at both A and B sites, and the same result occurred in isolated mice treated with 5‐HT_2C_ R antagonist SB243213 at B site of Htr2c RNA editing.

**Conclusions:**

The 5‐HT_2C_ R antagonist SB243213/5‐HT_2C_ R inverse agonist SB206553 recovered increased aggressive behavior of isolated BALB/c mice mediated by ADAR1 (p110) expression and Htr2c RNA editing.

## 1 INTRODUCTION

Social isolation has been widely recognized as one of the major factors resulting in the aggressive behavior of animals (Araki et al., 2016; Karpova, Mikheev, Marysheva, Bychkov, & Proshin, 2016; Rodríguez‐Arias et al., 2015; Takeda, Iwaki, Ide, Tamano, & Oku, 2012; White, Kucharik, & Moyer, 1991). However, thus far, the mechanism on how social isolation stress induces the increased aggressive behavior is still not clear. A fundamental understanding of how social isolation impacts the biological reactions in animals should be valuable to develop effective solutions to prevent or relieve the negative behavior resulting from social isolation. In a recent study, we showed that social isolation not only led to increased aggressive behavior (An, Chen, Yu, & Yin, 2016) but also impacted ADAR1 (p110) immune‐reactivity and protein expression in the brains of Kunming mice (Chen, An, Xu, & Yin, 2016). ADAR1 (Gene ID: ADAR) belongs to the ADAR family, which catalyzes the conversion of adenosine into inosine (A‐to‐I) in pre‐mRNA, while A‐to‐I RNA editing occurs at the A and B sites of Htr2c (Gene ID: 15560) RNA editing that has close relationships with the behavior of animals (Martin et al., 2013). In the central nervous system, serotonin is mediated by at least 14 receptor subtypes, in which 5‐HT_2C_R is the only receptor type to undergo A‐to‐I RNA editing, catalyzed by both ADAR1 and ADAR2, forming 24‐amino acid isoforms of the 5‐HT_2C_R (Werry, Loiacono, Sexton & Christopoulos, 2008). In addition, 5‐HT_2C_R is activated in the shock‐aggression mouse (Ennis et al., 2003). Juárez et al. (2013) showed that 5‐HT_2C_R can mediate the aggressive phenotype of TLX gene knockout mice. The RNA splicing and editing modulation of 5‐HT_2C_R function is relevant to aggression in VGV (full edited VGV isoform of 5‐HT_2C_R) mice (Martin et al., 2013). Furthermore, a novel 5‐HT_2C_R inverse agonist (S32212) suppresses aggressive behavior in mice (Dekeyne et al., 2012); the 5‐HT_2C_R antagonist is effective in reducing the aggressive behavior of the isolated mice (Juárez et al., 2013; Umukoro, Omogbiya, & Eduviere, 2013).

We hypothesized that social isolation stress impacts the expression of the RNA editing enzyme [ADAR1 (p110)] and Htr2c RNA editing, which is involved in an animals’ aggressive behavior. Another hypothesis is that any treatment that can bring ADAR1 (p110) expression and Htr2c RNA editing to the normal level can recover the animals’ aggressive behavior induced by isolation stress. To obtain evidence supporting these hypotheses, we quantified the changes in ADAR1 (p110) expression and Htr2c RNA editing for isolated BALB/c mice with aggressive behavior and evaluated the recovery of treatment with the 5‐HT_2C_R antagonist SB243213/5‐HT_2C_R inverse agonist SB206553 in this study.

## 2 MATERIALS AND METHODS

### 2.1 Animal groups and drug administrations

**Figure 1:**
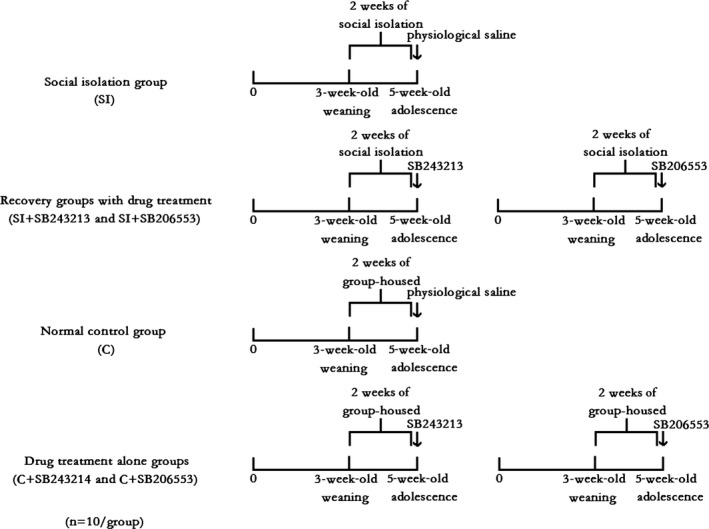
Groups of mice with and without 2 weeks of social isolation, followed by treatment with 5‐HT_2C_R antagonist SB243213/5‐HT_2C_R inverse agonist SB206553. Sixty healthy male BALB/c mice at the age of 21 days old were randomly divided into six groups, with 10 mice in each group. Each mouse was placed in a plastic cage (five mice per cage). The mice treated with physiological saline (20 ml/kg, i.p.) were individually housed for 2 weeks. The animals were labeled SI group (social isolation group, *n* = 10). In addition, the recovery of the behavioral deficit by 5‐HT_2C_ R antagonist SB243213/5‐HT_2C_ R inverse agonist SB206553 treatment (0.5 mg/kg, i.p.) was also examined. The treated groups were labeled SI + SB243213 group and SI + SB206553 group (recovery groups with drug treatments, *n* = 10/group). The age‐matched gregarious mice treated with physiological saline (20 ml/kg, i.p.) was labeled C group (normal control group, *n* = 10); meanwhile, the age‐matched gregarious mice treated with the 5‐HT_2C_ R antagonist SB243213/5‐HT_2C_ R inverse agonist SB206553 (0.5 mg/kg, i.p.) were labeled C+SB243213 and C+SB206553 (drug treatment alone groups, *n* = 10/group) groups, respectively

Sixty healthy male BALB/c mice at 21 days old (15 ± 5 g) were purchased from Dalian Medical University, Laboratory Center (Dalian, Liaoning, China), ID: 0003746. The animals were housed with the temperature (21 ± 1)°C, and the humidity was at (55 ± 5)%. The mice were fed with food and water ad libitum and randomly divided into six groups, with 10 mice in each group (Figure 1). Each mouse was placed in a plastic cage (Beijing Heli Technology Development Co. Ltd. China; 290 × 178 × 160 mm; 5 mice per cage). The mice were individually housed for 2 weeks, and treated with physiological saline (20 ml/kg i.p.). The mice were labeled the SI group (social isolation group, *n* = 10). In addition, according to the published literature (Navailles et al., 2013; Browne, Ji, Higgins, Fletcher, & Harvey‐Lewis, 2012) and our pilot study, recoveries of behavioral deficit by the 5‐HT_2C_R antagonist SB243213/5‐HT_2C_R inverse agonist SB206553 treatment (0.5 mg/kg, i.p.) were also examined, and the treated groups were labeled the SI+SB243213 group and the SI+SB206553 group (recovery groups with drug treatments, *n* = 10/group). The age‐matched gregarious mice treated with physiological saline (20 ml/kg, i.p.) were labeled the C group (normal control group, *n* = 10). Additionally, the age‐matched gregarious mice treated with the 5‐HT_2C_R antagonist SB243213/5‐HT_2C_R inverse agonist SB206553 (0.5 mg/kg, i.p.) were labeled the C+SB243213 and C+SB206553 groups (drug treatment alone groups, *n* = 10/group), respectively. All experimental procedures were approved by the Tab of Animal Experimental Ethical Inspection, Number: L20140021.

### 2.2 Resident‐intruder test

Aggressive ability was measured by observing a resident male mouse toward an unfamiliar male mouse intruder with no previous aggressive tendency. The unfamiliar male mouse with matched age and body weight was selected as the intruder. The mice were both placed into the local mouse (resident) home cage (M1, 29 × 17.8 × 16 cm; Beijing Heli Science & Technology Development Co. Ltd., Beijing, China) for 10 min, and their behaviors were videotaped. The latency to the first bite and duration of biting genital were evaluated. The duration of biting was calculated on genital (head, genital, and ventral area). The videos were evaluated by trained observers. The test was performed with light (20 LX) in the dark room from 9:00–11:00 a.m. (*n* = 10/group).

### 2.3 Immunohistochemistry

The mice were injected with 4% chloral hydrate for anesthesia (400 mg/kg, i.p.), followed by transcardial perfuse with 1% and 4% paraformaldehyde. The mouse brains were incubated in 4% paraformaldehyde for 24 hr, and then incubated in phosphate‐buffered saline (PBS) with 20% sucrose at 4°C overnight. Then, the slices were cut by a microtome‐cryostat at 16 μm thick. The selected slices were rinsed with PBS three times for 10 min each time and then incubated in 1% bovine serum albumin. Subsequently, the slices were covered with ADAR1‐Ab (p110) (1:100; Proteintech, USA) and then placed at 4°C overnight. The sections were rinsed with PBS three times for 10 min each time. After washing with PBS, the slices were treated with avidin–biotin complex at room temperature for 2 hr. The positive signals of ADAR1 (p110) were visualized with diaminobenzidine (DAB) for detection. Negative control slices were incubated with PBS without antibody. Image analysis for the quantification of the results was performed (*n* = 5/group).

### 2.4 Western blot analysis

Protein of amygdala was extracted using the extraction kit (Keygen Biotech, China). The protein concentration was assessed using the BCA protein assay kit (Keygen Biotech). Proteins (30 mg per sample) were denatured and then loaded onto 7.5% sodium dodecyl sulfate‐polyacrylamide (SDS) gels. Subsequently, the proteins were transferred to polyvinyl difluoride membranes blocked for 1 hr with 5% bovine serum albumin and then immunoblotted with the primary antibody ADAR1‐Ab (1:1,000; Proteintech). Subsequently, the membranes were washed with Tris‐buffered saline containing Tween 20 (TBST) and incubated with horseradish peroxidase‐labeled secondary antibody (anti‐goat 1:5,000; ZSJQ‐BIO Company, Beijing, China) for 2 hr at room temperature in a dark room. The infrared band signals were detected and quantified using Bio‐Rad (Hercules, CA, USA) gel analysis software. The membranes were then stripped using stripping buffer, washed in TBST, and probed with GADPH‐Ab (1:1,000; Beyotime Company, China). After washing again with TBST, the membranes were incubated with horseradish peroxidase‐labeled secondary antibody (anti‐mouse, 1:5,000; ZSJQ‐BIO Company, Beijing, China) and subsequently detected. ADAR1 (p110) protein expression was normalized by the internal control GADPH (*n* = 5/group).

### 2.5 Measurement of Htr2c RNA editing

**Figure 2:**
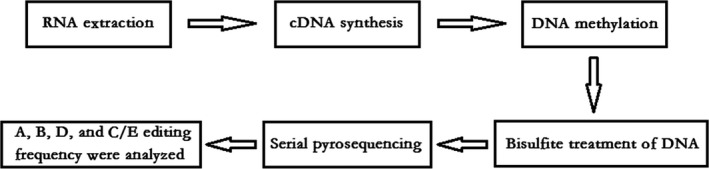
Outline of the procedure for measuring Htr2c RNA editing. Total RNA was extracted from the homogenized amygdala of the mice brain using the TRIzol method. Both RNA extraction and cDNA synthesis were performed using PrimeScript Hi‐Fide RT‐RCR Kit. Then, the prepared cDNA samples (*n* = 5 cDNA samples/group) were methylated by bisulfite treatment using Bisul‐Methylation Universal Kit. Then, PCR amplification of Htr2c was performed, with the used primers designed by PyroMark Assay Design 2.0 and synthesized by Hua Da Gene Company, as shown in Table 1. Subsequently, serial pyrosequencing measures were performed with the substrate mixture, enzyme mixture, and four types of dNTP added in the reaction system. Finally, pyrosequencing detector and Pyro Q‐CpG software were used to measure the frequency for A, B, D, and C/E editing sites

A previously described protocol (Tost & Gut, 2007) was used to measure Htr2c (Gene ID: 15560) RNA editing in this study. The entire experiment was performed in the laboratory (Biological Science & Technique Shanghai Zhuoli Co., Ltd., China). Figure 2 shows an outline of the procedure. Total RNA was extracted from homogenized amygdala of the mice brain using the TRIzol method (Invitrogen, America). Both RNA extraction and cDNA synthesis were performed using the PrimeScript Hi‐Fide RT‐RCR Kit (TAKARA Biotechnology Dalian CO., Ltd.). Then, the prepared cDNA samples (*n* = 5 cDNA samples/group) were methylated by bisulfite treatment using the Bisul‐Methylation Universal Kit (Qiagen, Germany). Next, the PCR amplification of Htr2c was performed, with primers designed by PyroMark Assay Design 2.0 and synthesized by the Hua Da Gene Company as shown in Table 1. Subsequently, serial pyrosequencing was performed with the substrate mixture, enzyme mixture, and four types of dNTP (Qiagen) in the reaction system. Finally, a pyrosequencing detector (PyroMark Q96 ID; Qiagen) and Pyro Q‐CpG software were used to measure the respective frequencies for A, B, D, and C/E editing sites.

**Table 1:** Primer of Htr2c

The primers	The sequence of the primers (5′–3′)
Htr2c—F	CGTCCATCATGCACCTCTG
Htr2c—R	GCCTTAGTCCGCGAATTGAA
Htr2c—S	TCGCTGGACCGGTAT

### 2.6 Statistical analyses

GraphPad Prism 5.0 (San Diego, CA, USA) and IBM SPSS Statistics 21.0 (Aramonk, NY, USA) were used for statistical analysis in the study. All data expressed as the means ± *SD* were analyzed using Tukey's post hoc test and LSD. A *t* test was used to analyze the variance for the groups with and without isolation, as well as the groups with and without drug treatment. Two‐way ANOVA was used to determine whether there is an interaction between social isolation and drug treatment (two independent variables) on aggressive behavior, ADAR1 expression, and RNA editing (dependent variable) among mice. The results of behavior analyses, immunohistochemistry, and Western blotting were analyzed using Tukey's post hoc test and LSD. The data for the 5‐HT_2C_R antagonist SB243213/5‐HT_2C_R inverse agonist SB206553 treatment were obtained from two separate analyses. The analysis of the RNA editing percentages has been analyzed using nonparametric methods and transformed as with percentages there is an imposed upper (100%) and lower (0%) limit, *p *<* *.05 was considered statistically significant (*n* = 10/group in resident‐intruder test, *n* = 5/group in immunohistochemistry staining, Western blot analysis, and Htr2c RNA editing measure, respectively).

## 3 RESULTS

### 3.1 Increased aggressive behavior by social isolation and its recovery after treatment with 5‐HT_2C_R antagonist/5‐HT_2C_R inverse agonist

**Figure 3:**
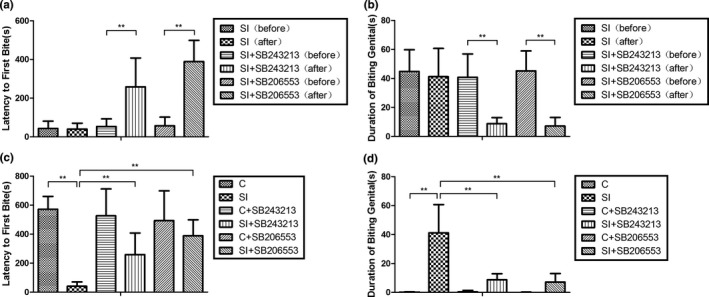
The 5‐HT_2C_ R antagonist SB243213/5‐HT_2C_ R inverse agonist SB206553 recovered the isolation‐induced increased aggressive ability in BALB/c mice. (a) Latency to first bite measured before and after the treatments with physiological saline/drugs for isolated BALB/c mice; (b) duration of biting genitals measured before and after the treatments with physiological saline/drugs for isolated BALB/c mice; (c) latency to first bite in all groups; and (d) duration of biting genitals in all groups. All data were statistically calculated as the means ± *SD*; **p <* *.01; (*n* = 10/group)

The analysis results of two‐way ANOVA showed that there was an interaction between social isolation and drug treatment (two independent variables) on aggressive behavior (Table 2). As shown in Figure 3a and b, the behavior tests before and after treatment with the 5‐HT_2C_R antagonist SB243213/5‐HT_2C_R inverse agonist SB206553 were performed to reduce the variability between mice. Compared with isolated mice prior to treatment, the mice treated with the 5‐HT_2C_R antagonist SB243213/5‐HT_2C_R inverse agonist SB206553 showed longer latency to first bite [SB243213: (isolated mice before the treatment: 53.45 ± 39.49; isolated mice after the treatment: 258.80 ± 148.96; *p *<* *.001); SB206553: (isolated mice before the treatment: 57.25 ± 44.74; isolated mice after the treatment: 389.15 ± 110.11; *p *<* *.001] as well as the shorter duration of biting genitals [SB243213: (isolated mice before the treatment: 40.80 ± 16.09; isolated mice after the treatment: 8.75 ± 4.22; *p *<* *.001); SB206553: (isolated mice before the treatment: 45.25 ± 13.75; isolated mice after the treatment: 7.15 ± 5.98; *p *<* *.001].

**Table 2:** *p*‐Values of the analysis by two‐way ANOVA

	Social isolation	Drug treatment	Social isolation × Drug treatment
Latency of first bit	.000	.013	.000
Duration of biting genitals	.000	.000	.000
BLA	.000	.045	.000
LaDL	.000	.002	.000
LaVL	.005	.001	.001
LaVM	.016	.001	.000
Western Blot	.014	.060	.080
Htr2c RNA editing	.039	.860	.006

LaDL, lateral amygdaloid nucleus, dorsolateral part; LaVM, lateral amygdaloid nucleus, ventromedial part; LaVL, lateral amygdaloid nucleus, ventrolateral part LaVL; BLA, basolateral amygdaloid.

The analysis results of two‐way ANOVA showed that there was an interaction between social isolation and drug treatment (two independent variables) on aggressive behavior, ADAR1 expression, and Htr2c RNA editing (dependent variable) among mice. Both isolation and drug treatment were associated with aggressive behavior, ADAR1 expression, and Htr2c RNA editing.

In addition, the behavior tests between social isolation and recovery groups were also performed to evaluate the recovery effect of the 5‐HT_2C_R antagonist SB243213/5‐HT_2C_R inverse agonist SB206553 on the enhanced aggressive behavior induced by isolation stress. The mice with 2 weeks of isolation showed decreased latency to the first bite compared to that for the control with age‐matched group‐housed mice (control group: 571.95 ± 88.70; 2‐week isolation group: 40.45 ± 29.64; *p *<* *.001). In addition, the duration for biting genitals for the isolated mice was much longer than that for the control, as shown in Figure 3c and d (control group: 0.10 ± 0.32; 2‐week isolation group: 41.20 ± 19.48; *p *<* *.001). These results indicated that social isolation increased the aggressive behavior of BALB/c mice. Most importantly, when the isolated mice were treated with the 5‐HT_2C_R antagonist SB243213/5‐HT_2C_R inverse agonist SB206553, the latency to first bite was longer than that of the isolated mice treated with physiological saline, as illustrated in Figure 3c [(2‐week isolation group: 40.45 ± 29.64; isolated mice treated with SB243213: 258.80 ± 148.96; *p *=* *.012) and (2‐week isolation group: 40.45 ± 29.64; isolated mice treated with SB206553: 389.15 ± 110.11; *p *<* *.001]. In addition, the isolated mice were treated with the 5‐HT_2C_R antagonist SB243213/5‐HT_2C_R inverse agonist SB206553, and the duration of biting genitals reduced compared to that for the isolated mice treated with physiological saline, as illustrated in Figure 3d [(2‐week isolation group: 41.20 ± 19.48; isolated mice treated with SB243213: 8.75 ± 4.22; *p *<* *.001) and (2‐week isolation group: 41.20 ± 19.48; isolated mice treated with SB206553: 7.15 ± 5.98; *p *<* *.001]. These results indicate that both SB243213 and SB206553 can relieve the enhanced aggressive behavior induced by isolation stress.

### 3.2 The decrease in ADAR1 (p110) immune‐reactivity and its recovery by treatment with 5‐HT_2C_R antagonist/5‐HT_2C_R inverse agonist

**Figure 4:**
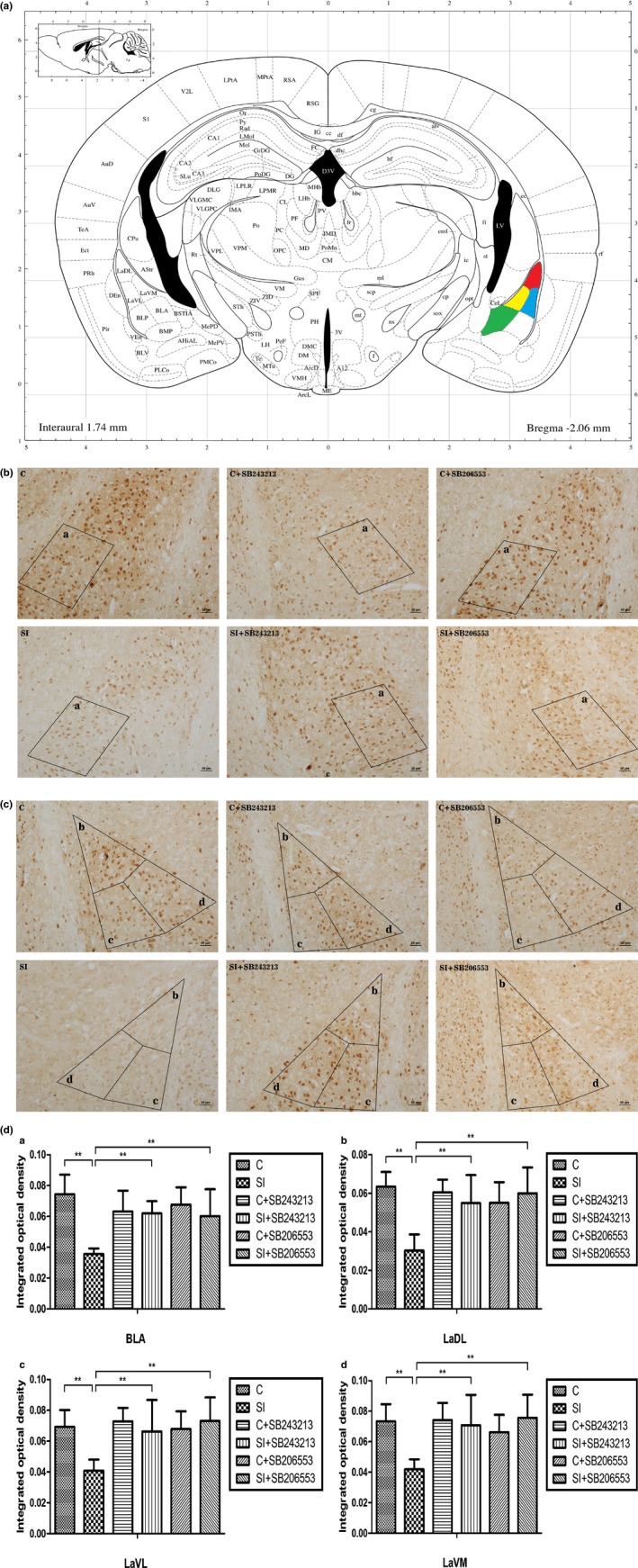
Decreased ADAR1 (p110) immune‐reactivity in amygdala of social isolated BALB/c mice and its recovery by the 5‐HT_2C_R antagonist SB243213/5‐HT_2C_R inverse agonist SB206553. (a) The brain regions were analyzed on the basis of the mouse brain atlas of Paxinos and Franklin (1997). a/Green represents BLA:basolateral amygdaloid nucleus; b/Red represents LaDL: lateral amygdaloid nucleus dorsolateral part; c/Blue represents LaVL: lateral amygdaloid nucleus ventrolateral part; d/Yellow represents LaVM: lateral amygdaloid nucleus ventromedial part. (b) Decreased ADAR1 (p110) immune‐reactivity‐positive signals in the BLA of the isolated mice and its recovery after treatment with the 5‐HT_2C_R antagonist SB243213/5‐HT_2C_R inverse agonist SB206553. Scale bar = 50 μm. (c) Decreased ADAR1 (p110) immune‐reactivity‐positive signals in LaDL, LaVL, and LaVM of the isolated mice and its recovery after treatment with the 5‐HT_2C_R antagonist SB243213/5‐HT_2C_R inverse agonist SB206553. Scale bar = 50 μm. (d) Statistical analysis of the integrated optical density of ADAR1 (p110) immune‐reactivity‐positive signals in the amygdala of the isolated mice and its recovery after treatment with the 5‐HT_2_ _C_R antagonist SB243213/5‐HT_2_ _C_R inverse agonist SB206553. All data were statistically calculated as the means ± *SD*; **p <* *.01; (*n* = 5/group)

The analysis results of two‐way ANOVA showed that there was an interaction between social isolation and drug treatment (two independent variables) on ADAR1 immune‐reactivity (Table 2). As shown in Figure 4b–d, 2 weeks of isolation led to obvious decreased immune‐reactivity‐positive signals of ADAR1 (p110) with the decreased optical density in amygdala of BALB/c mice compared to the age‐matched group‐housed mice. The following detailed data were obtained as follows: in the basolateral amygdaloid nucleus (BLA), control group: 0.074 ± 0.013; 2‐week isolation group: 0.036 ± 0.004; *p *<* *.001; in the lateral amygdaloid nucleus, dorsolateral part (LaDL), control group: 0.063 ± 0.008; 2‐week isolation group: 0.030 ± 0.008; *p *<* *.001; in the lateral amygdaloid nucleus, ventrolateral part (LaVL), control group: 0.069 ± 0.011; 2‐week isolation group: 0.041 ± 0.007; *p *<* *.001; in lateral amygdaloid nucleus, ventromedial part (LaVM), control group: 0.073 ± 0.011; 2‐week isolation group: 0.042 ± 0.007; *p *<* *.001.

Importantly, compared to the age‐matched isolated mice with treatment of physiological saline, isolated mice treated with the 5‐HT_2C_R antagonist SB243213/5‐HT_2C_R inverse agonist SB206553 showed higher ADAR1 (p110) immune‐reactivity‐positive signals in amygdala of BALB/c mice with the higher mean optical density. The following detailed data were obtained as follows: for SB243213‐treated isolated mice, in BLA, 2‐week isolation group: 0.036 ± 0.004; isolated mice treated with SB243213: 0.062 ± 0.008; *p *<* *.001; in LaDL, 2‐week isolation group: 0.030 ± 0.008; isolated mice treated with SB243213: 0.055 ± 0.015; *p *<* *.001; in LaVL, 2‐week isolation group: 0.041 ± 0.007; isolated mice treated with SB243213: 0.066 ± 0.020; *p *=* *.001; in LaVM, 2‐week isolation group: 0.042 ± 0.007; isolated mice treated with SB243213: 0.071 ± 0.020; *p *<* *.001. For isolated mice treated with SB206553, in BLA, 2‐week isolation group: 0.036 ± 0.004; isolated mice treated with SB206553: 0.060 ± 0.017; *p *<* *.001; in LaDL, 2‐week isolation group: 0.030 ± 0.008; isolated mice treated with SB206553: 0.060 ± 0.013; *p *<* *.001; in LaVL, 2‐week isolation group: 0.041 ± 0.007; isolated mice treated with SB206553: 0.073 ± 0.015; *p *<* *.001; in LaVM, 2‐week isolation group: 0.042 ± 0.007; and isolated mice treated with SB206553: 0.076 ± 0.015; *p *<* *.001.

These results indicated that the 5‐HT_2C_R antagonist SB243213/5‐HT_2C_R inverse agonist SB206553 decreased the immune‐reactivity of ADAR (p110) to normal levels in the amygdala of isolated mice.

### 3.3 The decrease in ADAR1 (p110) protein expression and the recovery by treatment with the 5‐HT_2C_R antagonist

**Figure 5:**
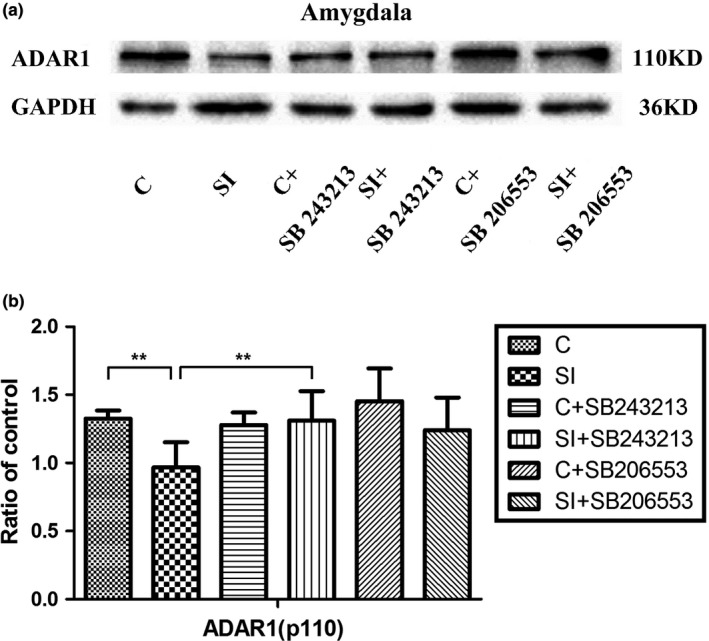
Decreased ADAR1 (p110) protein expression in the amygdala of socially isolated BALB/c mice and its recovery after treatment with the 5‐HT_2_ _C_R antagonist SB243213. (a) Decreased ADAR1 (p110) expression in the amygdala of isolated mice and its recovery after treatment with the 5‐HT_2_ _C_R antagonist SB243213. (b) Statistical analysis of the decreased ADAR1 (p110) in the amygdala of isolated mice and its recovery after treatment with the 5‐HT_2C_R antagonist SB243213. ADAR1 (p110) protein expression was normalized to the internal control GADPH. The data were expressed as the means ± *SD*; **p <* *.01 (C vs. SI); (*n* = 5/group)

The analysis results of two‐way ANOVA showed that there was an interaction between social isolation and drug treatment (two independent variables) on ADAR1 protein expression (Table 2). As shown in Figure 5a and b, the ADAR1 (p110) protein expression (control group: 1.33 ± 0.06; 2‐week isolation group: 0.97 ± 0.18; *p *=* *.005) in the amygdala was significantly decreased in the 2‐week social isolation group compared to age‐matched group‐housed control mice; however, isolated mice treated with 5‐HT_2C_R antagonist SB243213 showed obvious increased ADAR1 (p110) protein expression (2‐week isolation group: 0.97 ± 0.18; isolated mice treated with SB243213: 1.31 ± 0.22; *p *=* *.008) compared to that of the same age isolated mice with treatment of physiological saline. These results suggested that treatment with the 5‐HT_2C_R antagonist SB243213 returned the isolation‐induced decrease in ADAR1 (p110) protein expression to normal levels in the amygdala, significantly. Interestingly, isolated mice treated with the 5‐HT_2C_R inverse agonist SB206553 only showed increased tendency of ADAR1 (p110) protein expression compared to isolated mice without treatment. These results suggested that the effect of 5‐HT_2C_R antagonist SB243213 was much stronger than that of the 5‐HT_2C_R inverse agonist SB206553 for recovering the decreased ADAR1 (p110) protein expression in amygdala induced by isolation stress.

### 3.4 Treatment with 5‐HT_2C_R antagonist/5‐HT_2C_R inverse agonist decreased the percentage of Htr2c RNA editing at A and B sites in isolated BALB/c mice

The analysis results of two‐way ANOVA showed that there was an interaction between social isolation and drug treatment (two independent variables) on Htr2c RNA editing (Table 2). The RNA editing percentages were analyzed by nonparametric methods and transformed into percentages. The results showed an imposed upper (100%) and lower (0%) limit. As shown in Table 3, the percentage of Htr2c RNA editing at A and B sites of amygdala only showed a moderately increased tendency for 2‐week isolated mice compared to age‐matched group‐housed mice. Most importantly, the 5‐HT_2C_R inverse agonist SB206553 decreased the percentage of Htr2c RNA editing at both A (*p* = .027) and B (*p* = .014) sites to a level lower than that of the same age isolated mice with treatment of physiological saline. Interestingly, at the B site, the isolated mice treated with the 5‐HT_2C_R antagonist SB243213 showed a decreased percentage of Htr2c RNA editing compared to isolated mice of the same age and treated with physiological saline (*p* = .027), suggesting that both the 5‐HT_2C_R antagonist SB243213 and 5‐HT_2C_R inverse agonist SB206553 impacted Htr2c RNA editing of the isolated mice.

**Table 3:** Treatment with the 5‐HT_2C_ R antagonist SB243213/5‐HT_2C_ R inverse agonist SB206553 impacted percentage of Htr2c RNA editing at A and B sites of isolated BALB/c mice in amygdala on statistical analysis

Editing sites	C	SI	C + SB243213	SI + SB243213	C + SB206553	SI + SB206553
A site (%)	83.12 ± 2.10	85.78 ± 1.99*	85.51 ± 2.06	83.03 ± 1.84#	85.68 ± 0.75	82.06 ± 1.31+
B site (%)	67.23 ± 1.32	68.61 ± 2.39*	68.64 ± 3.20	65.32 ± 1.08#	68.06 ± 1.55	63.85 ± 1.32+
D site (%)	48.67 ± 3.91	43.51 ± 10.78	48.27 ± 1.45	47.12 ± 6.32	49.60 ± 2.67	48.37 ± 0.57
C/E site (%)	34.42 ± 3.33	33.38 ± 1.70	32.66 ± 1.16	32.20 ± 1.09	33.47 ± 0.77	31.80 ± 0.85

SI, social isolation.

The data were expressed as the means ± *SD*.

The above results were analyzed by *t* test, Post hoc testing of LSD and nonparametric methods. The *t* test results were *p =* *.297 (C vs. SI), #*p *=* *.041 (SI vs. SI + SB243213), ^+^ *p *=* *.028 (SI vs. SI + SB206553) at the A site, and *p =* *.250 (C vs. SI), ^#^ *p *=* *.028 (SI vs. SI + SB243213), ^+^ *p *=* *.043 (SI vs. SI + SB206553) at the B site. The hoc testing of LSD analyzed results was obtained as follows: at A site, *p =* *.143 (C vs. SI), ^#^ *p *=* *.055 (SI vs. SI + SB243213), ^+^ *p *=* *.012 (SI vs. SI + SB206553); at B site, *p =* *.299 (C vs. SI), ^#^ *p *=* *.019 (SI vs. SI + SB243213), ^+^ *p *=* *.001 (SI vs. SI + SB206553). The following nonparametric method analyzed results were obtained: at A site, *p = .221 (C vs. SI), ^#^ *p = *.086 (SI vs. SI + SB243213), ^+^ *p = *.027 (SI vs. SI + SB206553); at B site, *p = .327(C vs. SI), ^#^ *p = *.027 (SI vs. SI + SB243213), ^+^ *p = *.014 (SI vs. SI + SB206553) (*n* = 5/group).

## 4 DISCUSSION

### 4.1 Social isolation increased aggressive behavior

Previous studies have reported that social isolation stress is one of the major factors resulting in enhanced aggressive behavior. Social isolation led to enhanced aggressive behavior in C57BL/6j mice (Chang, Hsiao, Chen, Yu, & Gean, 2015; Dang et al., 2015; Ma, Jiang, Jiang, Wang, & Jia, 2011), ICR mice (Ouchi, Ono, Murakami, & Matsumoto, 2013; Yokota, Oshio, Moriya, & Takeda, 2016), ddY mice (Kawasaki et al., 2011; Vekovischeva, Verbitskaya, Aitta‐Aho, Sandnabba, & Korpi, 2007), and Swiss mice (Vekovischeva et al., 2007). The present findings provided evidence that 2‐week social isolation stress increased the aggressive behavior of BALB/c mice. These results are consistent with those of a previous study showing that isolation increased aggressiveness for both Kunming and BALB/c mice (An et al., 2016). These findings suggest that social isolation stress‐enhanced aggressive behavior in mice.

### 4.2 Effect of social isolation on Htr2c RNA editing

Among the protein‐coupled receptors, 5‐HT_2C_R is the only RNA editing receptor in the serotonin receptor family (Hackler, Airey, Shannon, Sodhi, & Sanders‐Bush, 2006; Martin et al., 2013), during which ADARs catalyzes five sites by converting adenosine (A) to inosine (I). Htr2c RNA editing is a dynamic process responsive to environmental challenges, including stress (Englander, Dulawa, Bhansali & Schmauss, 2005). However, little is known about how social isolation stress impacts Htr2c RNA editing. In the present study, we found that 2‐week isolation only led to moderately increased Htr2c RNA editing at A and B sites of amygdala in BALB/c mice (Table 3). Moreover, ADAR1 (p110) protein expression was decreased in the amygdala of isolated BALB/c mice, as illustrated in Figure 5. This finding is partially consistent with the results of a previous work, which demonstrated that social isolation induced abnormal ADAR1 (p110) protein expression in frontal cortex and hippocampus of isolated Kunming mice (Chen et al., 2016). The different changes in ADAR1 (p110) in response to social isolation stress could explain why different rodents have different genetics and environment interactions and explain the different brain areas regulating specific behaviors (An et al., 2016). The BALB/c mouse strain is a genetically distinct inbred strain with lower forebrain serotonin levels. Moreover, BALB/c mice showed spontaneously elevated anxiety and increased stress reactivity. Hackler et al. showed that in BALB/c mice, the majority of Htr2c mRNA is nonedited and encodes receptors with the highest constitutive activity and the highest agonist affinity. Additionally, when BALB/c mice were exposed to acute stress, site‐specific Htr2c pre‐mRNA editing increased, and function of mRNA encoding receptors decreased significantly (Hackler et al., 2006). These reports supported the use of BALB/c mice to explore the effects of social isolation on Htr2c RNA editing.

### 4.3 Effect of Htr2c RNA editing on aggressive behavior

VGV mice showed increased aggressive behavior associated with VGV editing patterns (Martin et al., 2013). However, the present findings did not provide direct evidence showing that Htr2c RNA editing is involved in increased aggressive behavior for isolated BALB/c mice. This lack of evidence may be due to moderate Htr2c RNA editing changes in response to the 2‐week isolation stress. The moderate changes in Htr2c RNA editing percentage at A and B sites may influence aggressive behavior by the effects on the postreceptor function of 5‐HT_2C_R (Martin et al., 2013), which will be addressed in future studies.

### 4.4 5‐HT_2C_R antagonist/5‐HT_2C_R inverse agonist recovered the increased aggressive behavior of isolated BALB/c mice mediated by ADAR1 expression and Htr2c RNA editing

Recent evidence suggests that both the 5‐HT_2C_R antagonist and 5‐HT_2C_R inverse agonist mitigate aggressive behavior. The 5‐HT_2A/C_R antagonist decreased aggressive behavior (Juárez et al., 2013; Umukoro et al., 2013). Furthermore, S32212, a combined 5‐HT_2C_R inverse agonist that also possesses 5‐HT_2A_R antagonist properties, suppressed aggressive behavior in mice (Dekeyne et al., 2012). Consistent with the above findings, we found that both the 5‐HT_2C_R antagonist SB243213 and the 5‐HT_2C_R inverse agonist SB206553 recovered the enhanced aggressive behavior of isolated BABL/c mice (Figure 3). However, the detailed molecular mechanism of how the 5‐HT_2C_R antagonist SB243213/5‐HT_2C_R inverse agonist SB206553 recovered abnormal aggressive behavior remains unknown. Compared with that of the age‐matched isolated mice treated with physiological saline, 5‐HT_2C_R inverse agonist SB206553 decreased the percentage of Htr2c RNA editing at both A and B sites, and isolated mice treated with 5‐HT_2C_R antagonist SB243213 showed a decreased percentage of Htr2c RNA editing at the B site. These findings demonstrated that the 5‐HT_2C_R antagonist SB243213/5‐HT_2C_R inverse agonist SB206553 impacted Htr2c RNA editing in isolated mice.

### 4.5 Proposed mechanism of how social isolation results in aggressive behavior

**Figure 6:**
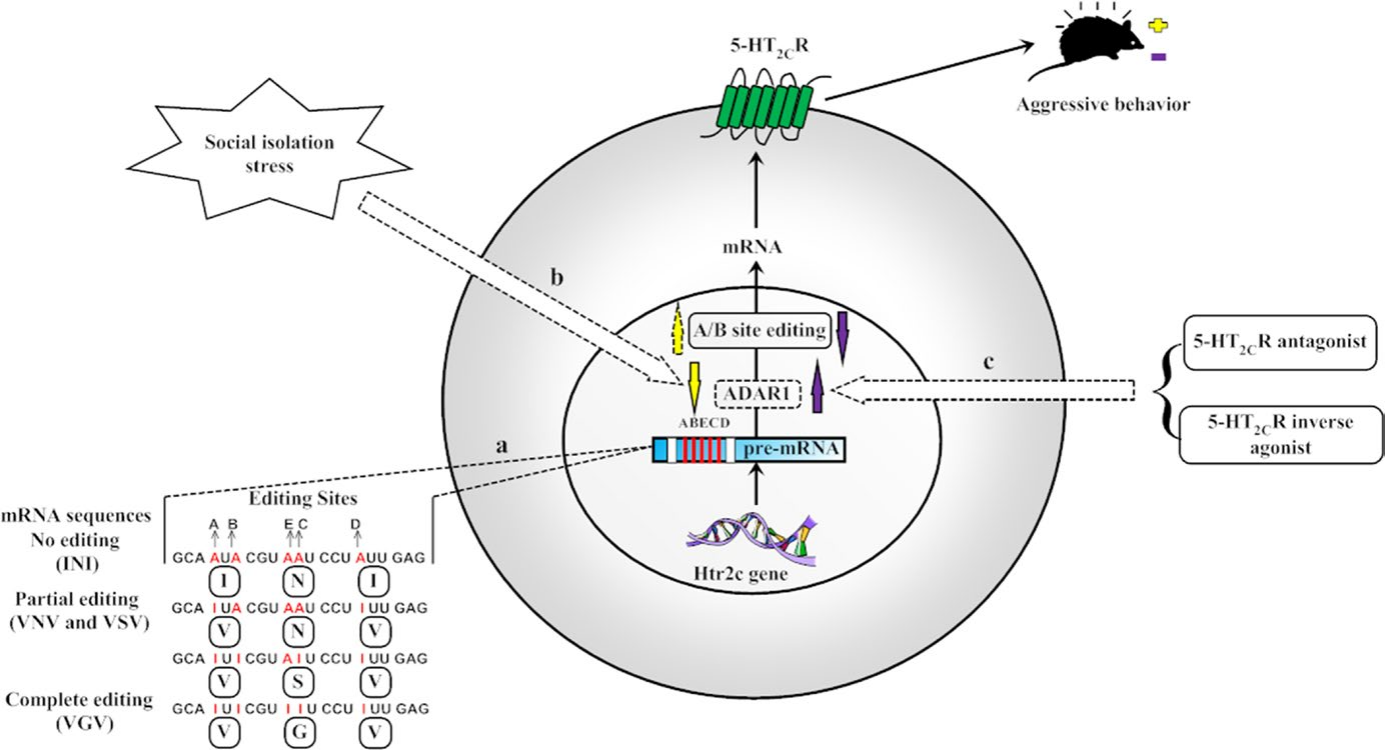
Hypothesis of how social isolation induces increased aggressive behavior mediated with ADAR1 (p110) and moderate changes in Htr2c RNA editing. (a) Htr2c RNA editing isoforms include no editing (INI), partial editing (VNV, VSV, and the other 28 amino acid isomers; Werry et al., 2008) and complete editing (VGV). (b) The isolated BALB/c mice with increased aggressive behavior showed the decreased protein expression of ADAR1 (p110) and moderate increased tendency of Htr2c RNA editing percentage at A and B sites of amygdala. (c) The treatment with 5‐HT_2C_R antagonist SB243213/5‐HT_2C_R inverse agonist SB206553 recovered the aggressive behavior of isolated mice and brought ADAR1 (p110) back to the normal level. Moreover, 5‐HT_2C_ R antagonist SB243213/5‐HT_2C_ R inverse agonist SB206553 decreased the percentage of Htr2c RNA editing at both A and B sites in isolated mice

Based on the findings of the present and previous studies, we proposed a mechanism explaining how social isolation results in aggressive behavior, as shown in Figure 6. As 5‐HT_2C_R plays an important role in aggressive behavior (Bigi, Maestripieri, Aloe, & Alleva, 1992; Navarro, Burón, & Martín‐López, 2008) and has five editing sites, A and B sites were edited by ADAR1 (Wang et al., 2004). In addition, Htr2c RNA editing has profound effects on receptor function. VGV mice showed an increased 5‐HT_2C_R density (Kawahara, Grimberg, & Teegarden, 2008 and Olaghere da Silva et al., 2010) and altered emotional behavior associated with the VGV editing patterns (Martin et al., 2013). Interestingly, for isolated mice, only ADAR1 (p110) showed an obvious decrease; however, Htr2c RNA editing showed a moderately increased tendency, suggesting that ADAR1 (p110) is more sensitive to isolation stress than Htr2c RNA editing. In addition, the moderate change in Htr2c RNA editing percentage at A and B sites may influence aggressive behavior by the effects on the postreceptor function of 5‐HT_2C_R (Martin et al., 2013). The hyperexcitability of BLA principal neurons caused abnormal aggressive behavior (Jing, Ping, Lu, Tao, & Zhen, 2017). The present findings showed that social isolation stress significantly decreased ADAR1 (p110) expression, including BLA, LaDL, LaVL, and LaVM, in the amygdala, as shown in Figure 4. Furthermore, the 5‐HT_2C_R antagonist and SB243213/5‐HT_2C_R inverse agonist SB206553 recovered the increased aggressive behavior of isolated BALB/c mice and returned the protein expression and immune‐reactivity of ADAR1 (p110) back to normal levels. Moreover, treatment with the 5‐HT_2C_R antagonist and SB243213/5‐HT_2C_R inverse agonist SB206553 significantly impacted the percentage of Htr2c RNA editing at A and B sites in the amygdala of isolated BALB/c mice, demonstrating that A and B sites of Htr2c RNA editing in isolated mice were more easily impacted by both the 5‐HT_2C_R antagonist SB243213 and the 5‐HT_2C_R inverse agonist SB206553 than that in age‐matched gregarious mice.

In conclusion, social isolation leads to not only increased aggressive behavior but also decreased ADAR1 (p110) expression in BALB/c mice. There were modest changes at the A and B sites of Htr2c RNA editing in isolated BALB/c mice; thus, it was more relevant to assess ADAR1 (p110) after the acute stress triggered by social stress using the resident‐intruder test. These findings provide further information to obtain a fundamental understanding of how social isolation impacts the physiological reactions that may lead to aggressive behavior in animals and explore an effective solution for the recovery of this behavior. In future studies, we will use conditional ADAR1 (p110) gene mutant mice and Htr2c RNA editing isoform mice (edited at A and B sites) to investigate the effects of the detailed molecular mechanism of Htr2c RNA editing on the postreceptor function of 5‐HT_2C_R and social isolation‐induced abnormal behavior.

## CONFLICT OF INTEREST

None declared.
